# TACE responser NDRG1 acts as a guardian against ferroptosis to drive tumorgenesis and metastasis in HCC

**DOI:** 10.1186/s12575-023-00199-x

**Published:** 2023-05-19

**Authors:** Bufu Tang, Yajie Wang, Jinyu Zhu, Jingjing Song, Shiji Fang, Qiaoyou Weng, Yang Yang, Jianfei Tu, Zhongwei Zhao, Minjiang Chen, Min Xu, Weiqian Chen, Jiansong Ji

**Affiliations:** 1 Key Laboratory of Imaging Diagnosis and Minimally Invasive Intervention Research, Lishui Hospital, School of Medicine, Zhejiang University, Lishui, 323000 China; 2 Department of Radiation Oncology, Key Laboratory of Cancer Prevention and Intervention, The Second Affiliated Hospital, College of Medicine, Zhejiang University, Hangzhou, 310058 China; 3 Department of Radiology, the Fifth Affiliated Hospital of Wenzhou Medical University, Lishui, 323000 China; 4 Department of Radiology, Second Affiliated Hospital, School of Medicine, Zhejiang University, Hangzhou, China

**Keywords:** Hepatocellular carcinoma (HCC), Transarterial chemoembolization (TACE), NDRG1, Prognosis, Immunotherapy

## Abstract

**Background:**

The treatment efficacy of transarterial chemoembolization (TACE) for hepatocellular carcinoma (HCC) varies widely between individuals. The aim of this study was to identify subtype landscapes and responser related to TACE, and further clarify the regulatory effect and corresponding mechanism of NDRG1 on HCC tumorgenesis and metastasis.

**Methods:**

The principal component analysis (PCA) algorithm was used to construct a TACE response scoring (TRscore) system. The random forest algorithm was applied to identify the TACE response-related core gene NDRG1 of HCC, and its role in the prognosis of HCC was explored. The role of NDRG1 in the progression and metastasis of HCC and functional mechanism were confirmed using several experimental methods.

**Results:**

Based on the GSE14520 and GSE104580 cohorts, we identified 2 TACE response-related molecular subtypes for HCC with significant differences in clinical features, and the TACE prognosis of Cluster A was significantly better than that of Cluster B (*p* < 0.0001). We then established the TRscore system and found that the low TRscore group showed a higher probability of survival and a lower rate of recurrence than the high TRscore group (*p* < 0.05) in both the HCC and TACE-treated HCC cohorts within the GSE14520 cohort. NDRG1 was determined to be the the hub gene associated with the TACE response of HCC and its high expression suggested a poor prognosis. Furthermore, The suppression of NDRG1 konckdown in tumorgenesis and metastasis of HCC was clarified in both vivo and vitro, which was importantly achieved through inducing ferroptosis in HCC cells, especially contributing to RLS3-induced ferroptosis.

**Conclusion:**

The constructed TACE response-related molecular subtypes and TRscores can specifically and accurately predict TACE prognosis for HCC. In addition, the TACE response-related hub gene NDRG1 may act as a guardian against ferroptosis to drive tumorgenesis and metastasis in HCC, which laid a new foundation for the development of new potential targeted therapy strategies to improve disease prognosis in HCC patients.

**Supplementary Information:**

The online version contains supplementary material available at 10.1186/s12575-023-00199-x.

## Background

Primary liver cancer (PLC) is the sixth most common malignancy and the third leading cause of cancer-related deaths worldwide, according to the World Health Organization (WHO) (https://gco.iarc.fr/today/fact-sheets-cancers) statistics. In 2020, more than 830,000 new deaths due to liver cancer occurred worldwide [1, 2], which has placed a great burden on the health care systems of various countries. Of these cancers, hepatocellular carcinoma (HCC) is the most common histological subtype of PLC (more than 90 percent) [3]. Due to the insidious onset, rapid progression and nonspecific clinical symptoms of HCC, most patients with HCC are already in the middle and advanced stages (BCLC stages B and C) when they see a doctor due to discomfort, they miss the optimal time window in which to receive curative treatment such as radical lobectomy and liver transplantation [4], and the survival prognosis is not optimistic. To improve the prognosis of such patients with HCC, transarterial chemotherapy embolization (TACE), local ablation, radiation therapy, molecularly targeted drug therapy, immunotherapy and other therapies have received widespread attention and exploration and have also demonstrated significant survival advantages in clinical applications [5].

TACE is the first-line recommended treatment for patients with HCC recommended by the BCLC staging system in the middle stage and is regarded as the "gold standard" for diagnosis and treatment. In actual clinical practice, TACE is also widely adopted as one of the treatments [6]. TACE embolizes the same vessel by injecting chemotherapeutic drugs after catheterization of the tumor-feeding artery to achieve a synergistic effect of strong cytotoxicity and ischemia, resulting in tumor necrosis [7]. Clinical evidence confirms that TACE can effectively prolong the survival time of HCC patients, and in patients who are sensitive to TACE treatment, the median overall survival time can reach 20–45 months [8]. However, the response of HCC patients to TACE varies greatly among individuals, and there is still a large proportion of patients who exhibit a poor or even no response to TACE [9, 10]. Many studies have attempted to further subdivide HCC patients and, thus, develop a variety of prognostic prediction models. Among them, the arterial embolization prognosis (HAP) score of liver cancer combined with tumor size, alpha-fetoprotein (AFP), bilirubin, and albumin values was used for prognostic evaluation [11]. Park et al. improved this further, adding tumor number as an evaluation factor as modified HAP-II (mHAP-II) [12]. However, most of these predictive models are HCC-specific rather than TACE-specific; thus, it is critical to develop a TACE-specific predictive method before TACE surgery to select candidates suitable for TACE therapy.

The liver has a unique immune microenvironment, and the tumor microenvironment (TME) in HCC prevents tumor cells from being recognized and eliminated by the immune system, which has a significant impact on tumorigenesis, progression, and response to treatment [13]. Immunotherapy is one of the most promising development directions in the field of tumor treatment, and its development in recent years has changed the approach to HCC treatment [14]. Immunotherapy kills cancer cells by activating the body's own immune system, with immune checkpoint inhibitors (ICIs) being the currently preferred immunotherapy strategy, and T-cell receptor programmed cell death receptor 1 (PD-1) and ligand programmed cell death-ligand 1 (PD-L1) are the main immune checkpoint molecules applied to anticancer immunotherapy [15–17]. However, the objective response rate of ICIs as HCC monotherapy is only approximately 15 to 20% [18], and immunotherapy resistance and poor responsiveness have been observed in more than 60% of reported cases of multiple cancer types [19]. It is worth noting that TACE has been found to aggravate the hypoxia of residual surviving tumors, which further leads to the formation of an immunosuppressive microenvironment through overexpression of VEGF, upregulation of PD-L1 expression, and inhibition of T-cell function [20, 21]. In addition, TACE has been shown in previous studies to release numerous tumor antigens and has potential synergies with ICIs [22]. Accordingly, exploring potential key genetic features that can effectively improve TACE reactivity in HCC and combining TACE with immunotherapy may be an effective way to achieve enhanced clinical efficacy of TACE and immunotherapy and improve the prognosis of patients.

In this study, we constructed a TACE response-related molecular subtype and TACE response scoring (TRscore) system for the specific and accurate prediction of TACE prognosis in HCC by performing differential gene expression analysis, consistent clustering analysis, principal component analysis (PCA) and random forest analysis of HCC patient mRNA information from the GSE14520 and GSE104580 cohorts in the GEO database. Immunotherapy and molecularly targeted drug therapy response prediction were carried out, laying the foundation for the clinical decision determination and individualized treatment plan implementation of HCC patients. In addition, we have identified core biomarkers, such as NDRG1 (associated with the TACE response of HCC), which are expected to become new therapeutic targets for HCC, providing new options for treatments with HCC. The flowchart of this project is shown in Figure S1.

## Materials and Methods

### Acquisition of Microarray and RNA Sequencing Data and Analysis of Differentially Expressed Genes (DEGs)

The microarray data of HCC were obtained from the GSE14520 and GSE104580 cohorts, both of which were downloaded from the GEO database (https://www.ncbi.nlm.nih.gov/geo/). The mRNA sequencing data were mainly downloaded from the ICGC database (https://dcc.icgc.org/) and TCGA database (https://www.cancer.gov/about-nci/organization/ccg/research/structural-genomics/tcga). The differentially expressed gene analysis of HCC patients was performed using the limma R package, with a corrected *P* value < 0.05 and |log2FoldChange|> 1 as the threshold. Venn diagrams were generated to obtain the DEGs shared between the GSE14520 and GSE104580 cohorts for subsequent analysis.

### Functional Enrichment Analysis

KEGG and GO functional enrichment analyses were performed through the Metascape online website (http://metascape.org) to explore the molecular biological functions, biological processes and regulatory gene pathways of TACE response-related DEGs. The results with an adjusted *p* value < 0.05 were considered statistically significant.

### Consistent Cluster Analysis

The R package "ConsensusClusterPlus" was used to perform a consistent clustering analysis. To select a range of k values (2, 3, 4…, 9), we wanted to cluster DEGs shared between the GSE14520 and GSE104580 cohorts and iterated 1000 times to ensure the stability of the classification. For each k, a consistency matrix was created after iteration, and, finally, the optimal matrix was selected based on the consistency distribution to identify the TACE response-related molecular subtypes of HCC. Kaplan–Meier (K-M) curve analysis was performed using the R package "survminer" to compare survival prognosis between subgroups.

### Calculation and Prediction Performance Evaluation of the TRscore

To quantify individual TACE responsiveness, we constructed a scoring system, the TRscore, to assess differences in TACE responsiveness in HCC patients. DEGs from different HCC response-related molecular subtypes were first normalized in all HCC patient samples in the GSE14520 cohort, and overlapping DEGs were analyzed by employing an unsupervised clustering method (K-means). Using the R packages "Rtsne" and "ggplot2" for the PCA algorithm, we defined TRscore with reference to a formula similar to that used in a previous study [23]: TRscore = ∑(PC1i + PC2i), where i is the TACE response correlation of HCC gene expression. The R packages "survival" and "survminer" were used to generate KM curves for predicting the clinical prognosis of the TRscore, and the R package "timeROC" was used to perform receiver operating characteristic curve (ROC) analysis by calculating the area under the ROC curve (AUC) to evaluate the prognostic predictive power of the TRscore.

### Gene Set Variation Analysis (GSVA) and Gene Set Enrichment Analysis (GSEA)

GSVA analysis was performed using the R package "GSVA" to evaluate differentially enriched pathways for DEGs. The immune function-related pathways used for GSVA were downloaded from the ImmPort database [24] (https://www.immport.org/home), and the metabolism-related pathways were obtained from the Molecular Signature Database (MSigDB) (http://software.broadinstitute.org/gsea/msigdb). Adjusted *p* values < 0.05 were considered statistically significant. GSEA was performed using the R package "clusterProfiler" to explore the enrichment significance of DEGs among tumor metabolism-related pathways, with the screening conditions |Normalized Enrichment Score (NES)|> 1, nominal (NOM) p value < 0.05 and FDR q-value < 0.25.

### Prediction of Drug Sensitivity

The association of TRscore with molecularly targeted drug sensitivity in the GSE14520 and HCC-TACE cohorts was predicted by the Genomics of Cancer Drug Sensitivity (GDSC) database (https://www.cancerrxgene.org/). Drug response was assessed using the R package "pRophetic" prediction, where ridge regression was used to estimate the semi-inhibitory concentration (IC50) of the high versus low TRscore populations, and the accuracy of the prediction was estimated by tenfold cross-validation.

### Construction of the Nomogram and Performance Evaluation

Univariate and multivariate Cox regression analyses were performed using the R package "survival" with TRscore and traditional clinical prognostic factors as variables to identify independent predictors of TACE prognosis in HCC. Based on the independent prognostic factors screened by Cox analysis, the R package "rms" was used to generate a nomogram for predicting survival outcomes, and a calibration plot was generated to compare the predictive power of the nomogram for survival probability.

### Random Forest Analysis

The R package "random Forest SRC" was used to randomly select n samples from the GSE14520 cohort with the Bootstrap sampling method to generate n survival trees and then randomly select a subset of covariates at each node of the tree as candidate variables for splitting. The OOB error rate of out-of-bag (OOB) samples and the error rate of the bag under different parameter combinations were calculated, and the optimal parameter combination that minimized the overall error rate of the random forest was then determined. We calculated the variable importance (VIMP) for each parametric variable in a parametric combination to assess the predictive power of the predictor over the outcome variable.

### Cell Culture

The human-derived liver cancer cell lines SK-HEP1 and HCC-LM3 were cultured in a constant-temperature incubator containing 5% CO_2_ and a relative air saturation humidity of 95% at 37 °C.

### Western Blot

Proteins were obtained from SK-HEP1 and HCCLM3 cells using RIPA lysis buffer (Invitrogen, Carlsbad, USA) supplemented with 1% protease and phosphatase inhibitors (Thermo, Shanghai, China). The separation of protein samples of different molecular weights was achieved by 10% sodium dodecyl sulfate–polyacrylamide gel electrophoresis (SDS–PAGE). Separated proteins were electrotransferred onto polyvinylidene fluoride (PVDF) membranes (Invitrogen), blocked with 5% nonfat milk for 90 min at room temperature, and incubated with primary antibody overnight at 4 °C, followed by incubation with an HRP-conjugated secondary antibody. The combined secondary antibody was incubated for 2 h at room temperature. Signals were detected, and blots were imaged using an iBright FL1500 Intelligent Imaging System (Invitrogen).

### Lentiviral Infection and Selection of Stable Cells

The SK-HEP1 and HCC-LM3 cell lines to be infected were plated in a 6-well culture dish at an appropriate density. After 24 h of culture, an appropriate amount of virus was added to the culture dish according to the multiplicity of infection (MOI) value of the target cells and the concentration of the virus solution. The solution was mixed and placed in a constant-temperature CO_2_ incubator at 37 °C for 24 h. After 48–72 h of cell infection, the cells were incubated with DMEM complete medium containing 1 ng/ml puromycin. After the cells grew steadily, cellular proteins were collected to detect the knockdown efficiency of the target gene.

### CCK8 and EdU Assays to Assess Cell Proliferation

The CCK-8 assay was as follows: SK-HEP1 and HCC-LM3 cells were seeded in 96-well plates at 3000 cells per well and 100 μL of DMEM. After culturing the cells for 0, 24, 48, and 72 h, the medium was replaced with fresh medium supplemented with 10% CCK-8 reagent, and the cells were incubated for an additional 2 h at 37 °C. The absorbance of the cells at 450 nm was measured using a microplate reader (Bio–Rad, Berkeley, CA, USA). For the EdU assay, SK-HEP1 and HCC-LM3 cells were incubated for 24 h. Then, 100 μL of 50 μM EdU reagent was added to each well and incubated for 2 h. Cells were then fixed with 4% paraformaldehyde for 30 min at room temperature and incubated with Apollo staining solution and DAPI for 30 min. Fluorescence microscopy was used to detect cell proliferation.

### Live/Dead Cell Staining Experiment (Calcein-AM/PI)

Then, 20 μl of Calcein-AM and 5 μl of PI solution were added to 10 ml of DMEM without FBS, mixed well and protected from light. The cultured cells were washed 2–3 times with prewarmed PBS at 37 °C, and 1 ml of calcein-AM and PI mixed solution was then added. The cells were incubated in a constant-temperature CO_2_ incubator at 37 °C for 10–15 min, and the mixed solution was aspirated and discarded. After the cells were washed 3–5 times, 1 ml of DMEM complete medium was added, and the cells were observed under a fluorescence microscope.

### Transwell Cell Migration Assay

Cell migration assays were performed using Corning Costar Transwell chambers. Ahead of the transwell assay, SK-HEP1 and HCC-LM3 cells were treated with mitomycin C (10 μg/ml) for 1 h to suppress cell proliferation to eliminate the effect of proliferation on migration. Serum-free DMEM (Gibco, NY, USA) was added to the upper chamber, and DMEM supplemented with 10% fetal bovine serum was added to the lower chamber. The cells were trypsinized, centrifuged and resuspended in the upper-chamber medium at a density of 2.5 × 10^5^ cells per well. After 48 h of incubation at 37 °C, the unmigrated cells on the upper side of the upper chamber were removed with a cotton swab, and the cells that migrated to the lower side of the upper chamber were stained with 0.1% crystal violet and placed under a microscope for image acquisition and analysis.

### Cellular Immunofluorescence

After rinsing the slides with PBS, the cells were fixed with 4% paraformaldehyde solution at room temperature for 30 min, and PBS solution containing 0.5% Triton X-100 was then added for 30 min to permeabilize the cells. Next, blocking solution was added and incubated at room temperature for 60 min. Then, primary antibody diluent was added and incubated overnight at 4 °C on a shaker. On the second day, the secondary antibody diluent was added and incubated in the dark for 60 min, and 500 μl of 10 µg/ml DAPI solution was then added and incubated for 10–15 min. Anti-fluorescence quenching mounting medium was used to seal the slide under dark conditions, and the slides were placed under a confocal fluorescence microscope for image acquisition and analysis.

### Immunohistochemical Staining

The paraffin samples of tumor tissue from HCC patients and the corresponding normal tissue adjacent to the tumor were sectioned at 4 μm and dried. The paraffin sections were sequentially subjected to xylene dewaxing through immersion in ethanol dexylene, and the sections were then immersed in 3% hydrogen peroxide (H_2_O_2_) solution and incubated for 20–30 min. The sections were blocked at room temperature for 1 h, and the primary antibody solution was then added dropwise and incubated overnight at 4 °C. After washing with PBS, the sections were incubated with avidin/biotinylated horseradish peroxidase for 2 h and then reacted with 3,3'-diaminobenzidine (DAB) as a chromogen for immunohistochemical staining. The slides were covered with neutral resin and placed in a fume hood to air dry overnight. After drying, the sections were observed under a microscope to obtain immunohistochemical images for analysis.

### Statistical Analysis of Data

The experimental data were statistically analyzed using GraphPad Prism 9 (version 8.0.2) and R software (version 3.6.3), and the results are presented as the mean ± standard error of the mean (SEM). Adjusted P values < 0.05 were considered statistically significant. All experiments in this subject were independently repeated 3 times.

## Results

### Determination of TACE Responsiveness-related Molecular Subtypes of HCC

**Fig. 1:**
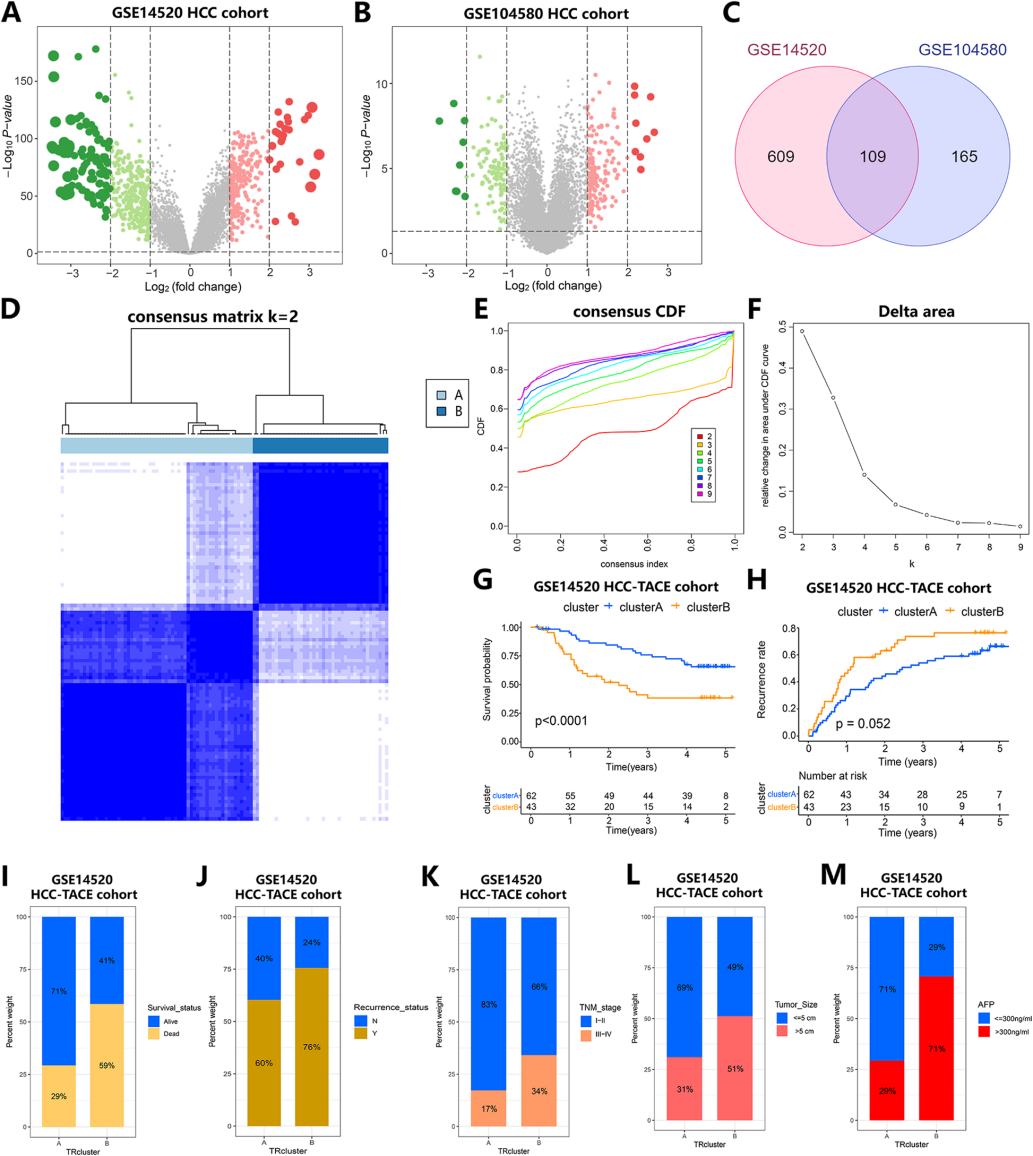
Determination of TACE responsiveness-related molecular subtypes of HCC and correlation with clinical features.** A** Volcano plot showing differential gene expression between paracancerous tissues and HCC tissues in the GSE14520 cohort. **B** Volcano plot showing differential gene expression between the HCC cohort that responded to TACE treatment and the HCC cohort that did not respond to TACE treatment within the GSE104580 cohort. Green indicates downregulated differential genes, and red indicates upregulated differential genes (|log FC|> 1 and adjusted *p* < 0.05). **C** Venn diagram showing DEGs shared between the GSE14520 and GSE104580 cohorts. **D** Consensus clustering matrix for k = 2. **E** Consistent clustered cumulative distribution function (CDF) curves for k values from 2 to 9. **F** CDF delta area curve. The horizontal axis represents the number of categories k, and the vertical axis represents the relative change in the area under the CDF curve. **G** The K-M curve shows the difference in survival between Cluster A and Cluster B. **H** The K-M curve shows the difference in recurrence between Cluster A and Cluster B. **I** Differences in survival between Cluster A and Cluster B. **J** Differences in recurrence status between Clusters A and B. **K** TNM staging between Clusters A and B. **L** Tumor size distribution characteristics between Clusters A and B. **M** AFP differences between Clusters A and B

To analyze the TACE response-related genes of HCC, we first performed a differential analysis of the HCC cohort (paracancer and HCC samples) of the GSE14520 cohort and the TACE-treated cohort (TACE responders and nonresponders) from the GSE104580 cohort. The volcano plot shows the distribution of DEG expression in these two cohorts (Fig. 1A, B), and we applied the Venn plot to intersect the HCC-related DEGs and TACE-reactivity-related DEGs. Finally, we obtained 109 shared DEGs, and we identified these 109 DEGs as genes related to the TACE response of HCC (Fig. 1C). Subsequently, we used the R package "ConsensusClusterPlus" to establish a consistent clustering of these 109 DEGs in the HCC-TACE cohort within the GSE14520 cohort, and the results showed the clustering effect when HCC samples were divided into two subtypes (Cluster A, Cluster B). This approach was optimal, with better intrasubtype consistency and stability (Fig. 1D-F). Overall survival (OS) results when analyzing the prognosis associated with these two clusters showed that Cluster A had a better survival prognosis than Cluster B (*p* < 0.0001) (Fig. 1G), while recurrence between Clusters A and B was not significantly different (*p* > 0.05) (Fig. 1H). When further examining the clinical characteristics between Clusters A and B, we found that Cluster A exhibited better survival than Cluster B (Fig. 1I) and a lower proportion of relapsed states than Cluster B (Fig. 1J). In addition, 83% of patients in Cluster A were in TNM stages I-II, while 34% of patients in Cluster B were in TNM stages III-IV (Fig. 1K). In Cluster A, 69% of patients had tumors ≤ 5 cm and 71% had AFP ≤ 300 ng/ml, while 51% of Cluster B patients had tumors > 5 cm and 71% had AFP > 300 ng/ml (Fig. 1L, M). The above results indicated that the TACE response-related molecular subtypes of HCC were closely related to the clinical characteristics of HCC patients, and the prognosis of Cluster A was significantly better than that of Cluster B.

### The Predictive Value of the TRscore Scoring System Based on TACE Responsiveness-related Molecular Subtypes of HCC in the Prognosis of TACE

**Fig. 2:**
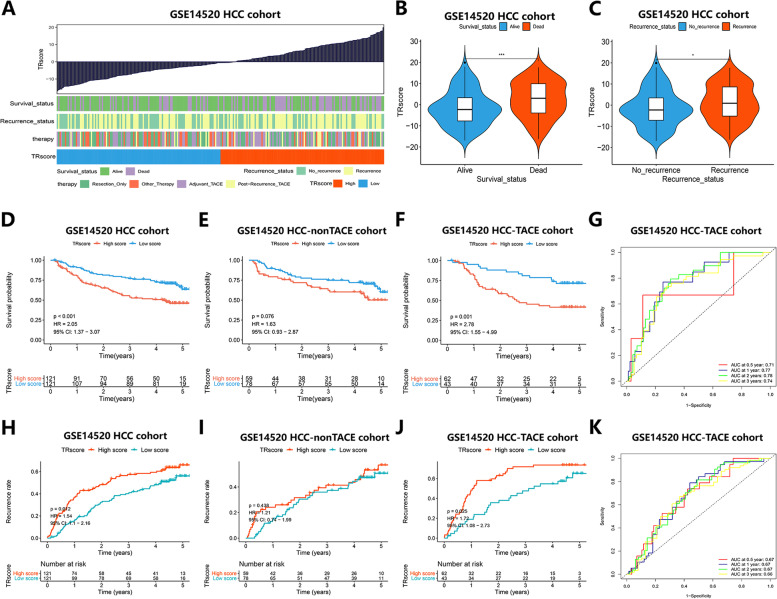
Predictive value of the TRscore in TACE prognosis in the GSE14520 cohort.** A** Corresponding clinical characteristics of patients with different TRscores. **B** Violin plot showing the correlation between survival status and TRscore (***p < 0.001). **C** Violin plot showing the correlation between relapse status and TRscore (*p < 0.05). **D-F** K-M curves showing the difference in survival between the high TRscore group and the low TRscore group in all HCC cohorts (**D**), HCC-nonTACE cohorts (**E**) and HCC-TACE cohorts (**F**). **G** ROC curves showing the survival prediction accuracy of the TRscore in the HCC-TACE cohort. **H-J** K-M curve showing the difference in recurrence rate between the high TRscore group and the low TRscore group in all HCC cohorts (**H**), HCC-nonTACE cohorts (**I**) and HCC-TACE cohorts (**J**). **K** ROC curves showing the recurrence prediction accuracy of the TR score in the HCC-TACE cohort

To further quantify individual TACE responsiveness, we constructed a TRscore scoring system using PCA to assess differences in TACE responsiveness in HCC patients. The median TRscore was used as the cutoff value to divide the patients into a high TRscore group and a low TRscore group, and the corresponding survival status, disease recurrence, and treatment differences of HCC patients with different TRscores were analyzed (Fig. 2A). When analyzing the correlation between the TRscore and the survival status and disease recurrence of HCC patients, we found that the TRscore level of the survival group was significantly lower than that of the death group (*p* < 0.001) (Fig. 2B), and the tumors did not recur. The group also showed a lower TRscore (*p* < 0.05) than the group with relapse (Fig. 2C).

To verify whether the TRscore can specifically predict the TACE prognosis of HCC, we performed prognostic survival analysis in all HCC, HCC-nonTACE and HCC-TACE cohorts within the GSE14520 cohort. The results showed that there was no significant difference in survival and recurrence rates between the high- and low-TRscore groups in the HCC-nonTACE cohort (*p* > 0.05) (Fig. 2E, I), while the low-TRscore group of the TACE cohort showed a significant survival advantage over the high-TRscore group (*p* < 0.05) (Fig. 2E, F), and the recurrence rate was significantly lower than that in the corresponding high-TRscore group (*p* < 0.05) (Fig. 2I, J). When evaluating the prognostic prediction performance of the TRscore in the HCC-TACE cohort, the ROC results showed that the AUC for survival prediction reached 0.71, 0.77, 0.78, and 0.74 at 0.5, 1, 2, and 3 years, respectively (Fig. 2G), while the AUC for recurrence prediction was 0.67, 0.67, 0.67, and 0.66 at 0.5, 1, 2, and 3 years, respectively (Fig. 2K), suggesting that the TRscore has good predictive specificity and sensitivity. The above results indicate that the TRscore is specific for predicting the prognosis of the HCC population receiving TACE treatment. The TACE prognosis of patients with a low TRscore was significantly better than that of patients with a high TRscore, and the prediction performance was excellent.

### Correlation of TRscore with Clinical Characteristics and Predictive Role of TACE Responsiveness

**Fig. 3:**
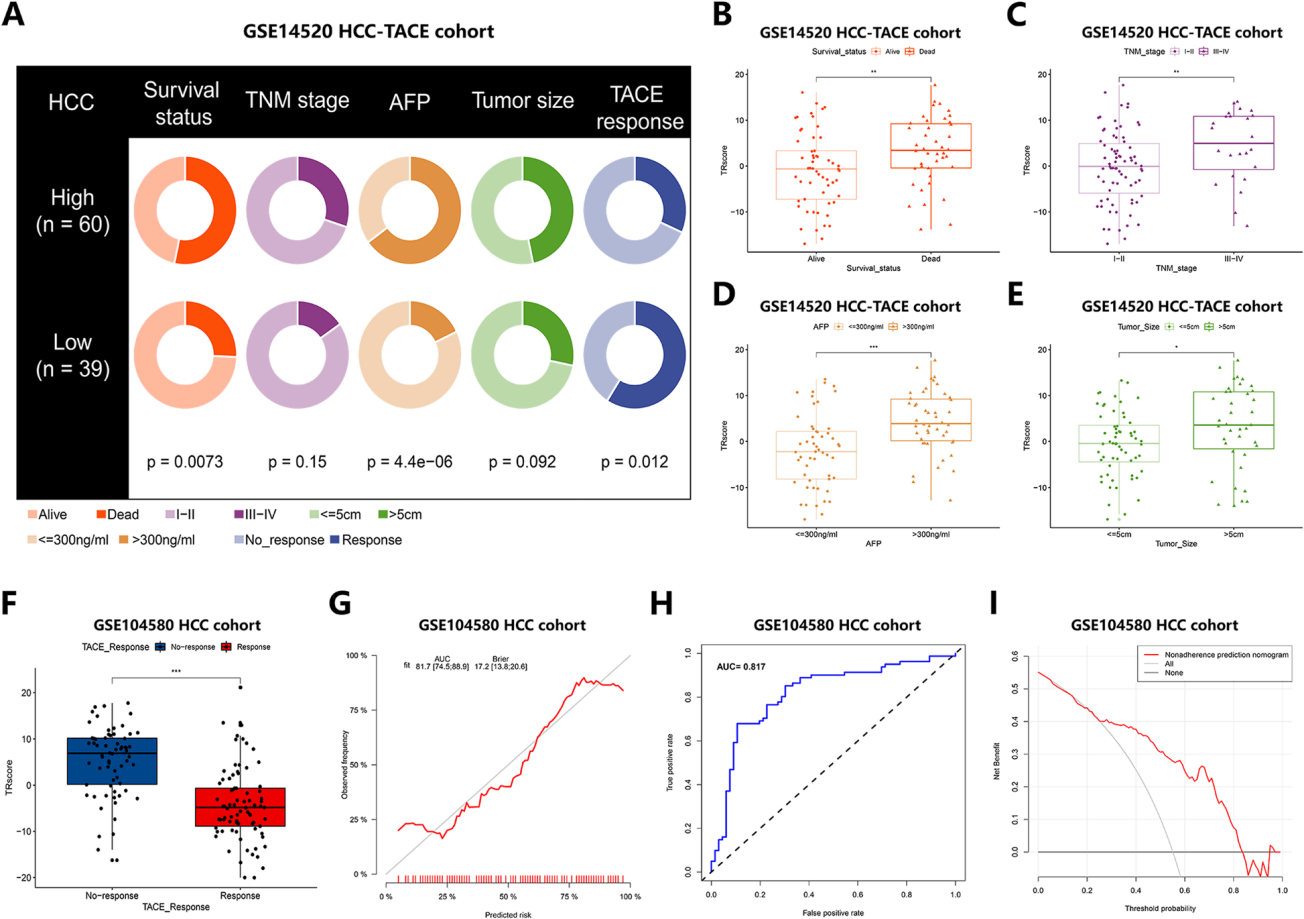
Correlation of TRscore with clinical characteristics in the HCC-TACE cohort of the GSE14520 cohort. **A** Donut-shaped pie chart showing differences in clinical characteristics between the high and low TRscore groups. **B-E** Boxplots showing that survival status (**B**), TNM stage (**C**), AFP value (**D**), and tumor size (**E**) correlated with TRscore (*p < 0.05, **p < 0.01, ** *p < 0.001). **F** Boxplots showing the difference in the distribution of TR scores between TACE responders and TACE nonresponders in the HCC-TACE cohort of the GSE104580 cohort (*p < 0.05, ***p < 0.001). **G** Concordance curves showing concordance of correlation predictions. **H** AUC values of ROC curves assess the reliability of correlation prediction. **I** DCA curves showing clinical benefit predicted by correlation

To elucidate the correlation between TRscore and clinical features, we analyzed the differences in the distribution of survival status, TNM stage, AFP value, tumor size, and TACE reactivity of the HCC-TACE cohort within the GSE14520 cohort under different TRscores (Fig. 3A). Further boxplot results showed that HCC patients who died and had a TNM stage III-IV, AFP > 300 ng/ml, and tumor size > 5 cm tended to have higher TRscores (*p* < 0.05) (Fig. 3B-E). To evaluate the predictive performance of the TRscore for TACE response in HCC, we used the GSE104580 cohort for validation, and the results showed that the TACE responder group exhibited a tendency toward lower TRscores (*p* < 0.05) than the TACE nonresponder group (*P* < 0.05) (Fig. 3F). The corresponding predicted AUC value was 0.817 (Fig. 3G, H). We also performed DCA to assess the value of the TRscore in clinical decision-making. The DCA curve results suggested that the TRscore had good predictive performance (F ig. 3I). The above results suggest that the TRscore is closely related to clinical disease characteristics such as survival status, TNM stage, AFP value, tumor size, and TACE reactivity. Patients with high TRscores have more severe disease characteristics and worse TACE prognoses than patients with low TRscores.

### Correlation of TRscore with Differential Expression of the Tumor Immune Microenvironment and Immune Checkpoints

**Fig. 4:**
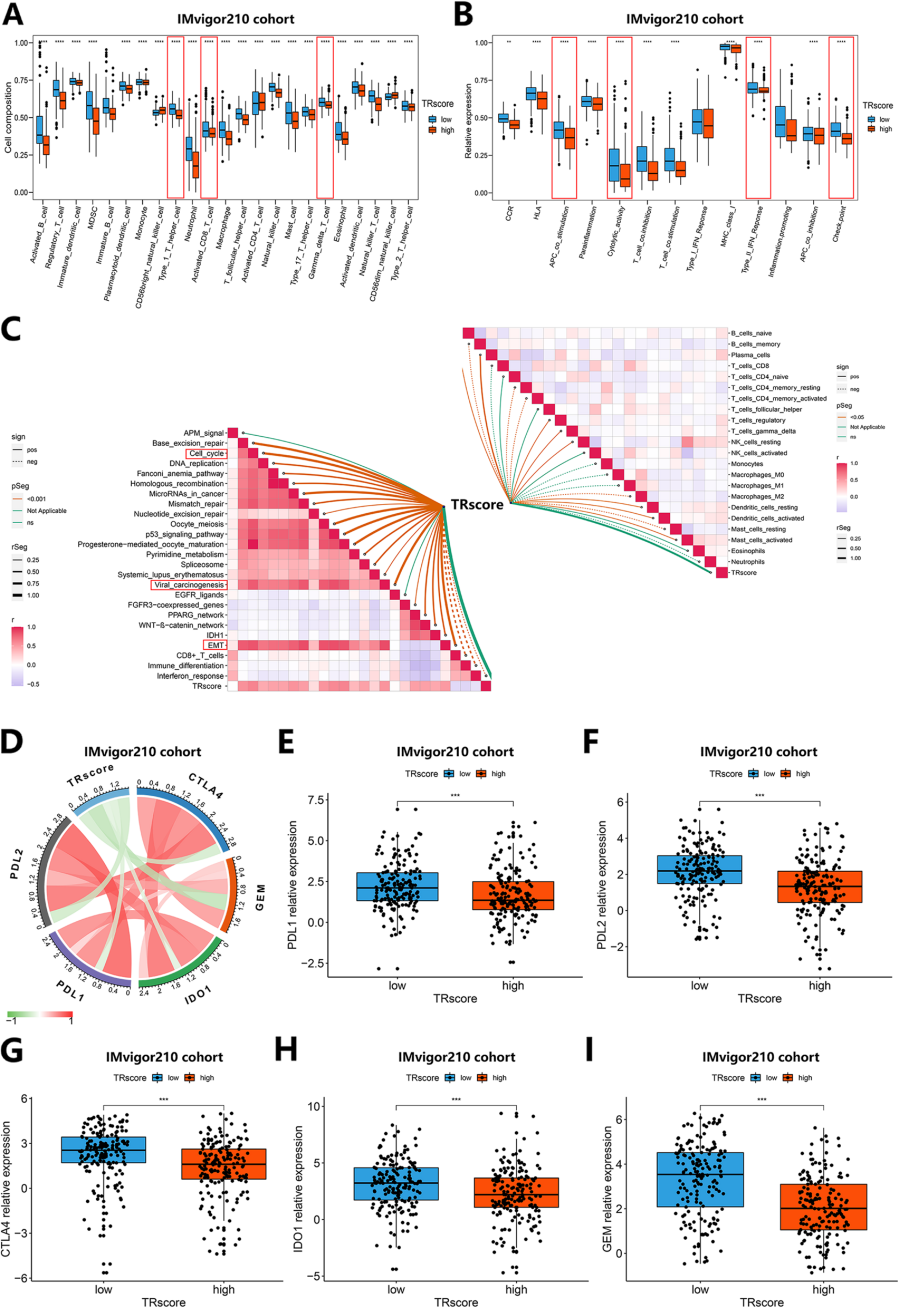
Correlation between TRscore and the tumor immune microenvironment in the IMvigor210 cohort.** A** Differences in immune cell infiltration between the high- and low-TRscore groups (****p < 0.0001). **B** Differences in immune function between the high- and low-TRscore groups (**p < 0.01, ****p < 0.0001). **C** Correlations between TRscore levels, immune cells and tumor-related regulatory pathways. **D** Correlation between TRscore and the expression of common immune checkpoints. **E-I** Differences in PDL1 (**E**), PDL2 (**F**), CTLA4 (**G**), IDO1 (**H**) and GEM (**I**) expression between the high and low TRscore groups (***p < 0.001)

Previous studies have shown that TACE treatment affects the formation and alteration of the tumor immune microenvironment [20, 21] and has potential synergistic effects with immunotherapy [22]. Therefore, we analyzed the association between the TRscore and the tumor immune microenvironment through an independent immunotherapy cohort, IMvigor210. We first explored whether the TRscore is related to the degree of immune cell infiltration and immune function in the tumor microenvironment. The results showed that there were significant differences in the infiltration levels of most immune cells in the different TRscore groups. Among them, the activated CD8 + T cells, type I CD4 + helper T cells (Th1 cells) and γδ T cells in the low TRscore group interacted with tumor-killing immune cells. The infiltration level of cells was significantly higher than that in the high-TRscore group (*p* < 0.0001) (Fig. 4A). In addition, compared with the high TRscore group, the low TRscore group showed stronger antigen-presenting cell (APC) costimulation, type II IFN response and immune cytolytic activity (CYT), which can induce or enhance antitumor immunity and functional levels. The low-TRscore group expressed higher levels of immune checkpoints than the high-TRscore group (*p* < 0.0001) (Fig. 4B). Tumor-related metabolic pathways that play a promoting role in tumor development, such as the cell cycle, viral oncogenicity, and epithelial-mesenchymal transition (EMT), were also confirmed to be positively correlated with TRscore levels (*p* < 0.05) (Fig. 4C). The above results suggest that the TRscore is closely related to immune cell infiltration and immune function in the tumor immune microenvironment. The low TRscore group showed stronger antitumor immunity than the high TRscore group, and the effect of immunotherapy may be better, while the high TRscore group showed stronger antitumor immunity than the high TRscore group. Groups tended to have higher expression levels of tumor-promoting signaling pathways, which were favorable for tumor development.

Considering that immune checkpoint inhibition is the preferred strategy for immunotherapy, we assessed the correlation between TRscore levels and common immune checkpoints (Fig. 4D). The results showed that compared with the high-TRscore group, the low-TRscore group exhibited higher expression levels of PDL1, PDL2, CTLA4, IDO1 and GEM (Fig. 4E-I), suggesting that the low-TRscore group was a more suitable candidate for immunotherapy and that more significant therapeutic effects could be obtained from immune checkpoint-targeted therapy.

### Independent Prognostic Ability Assessment of the TRscore and Construction of the Nomogram Model

**Fig. 5:**
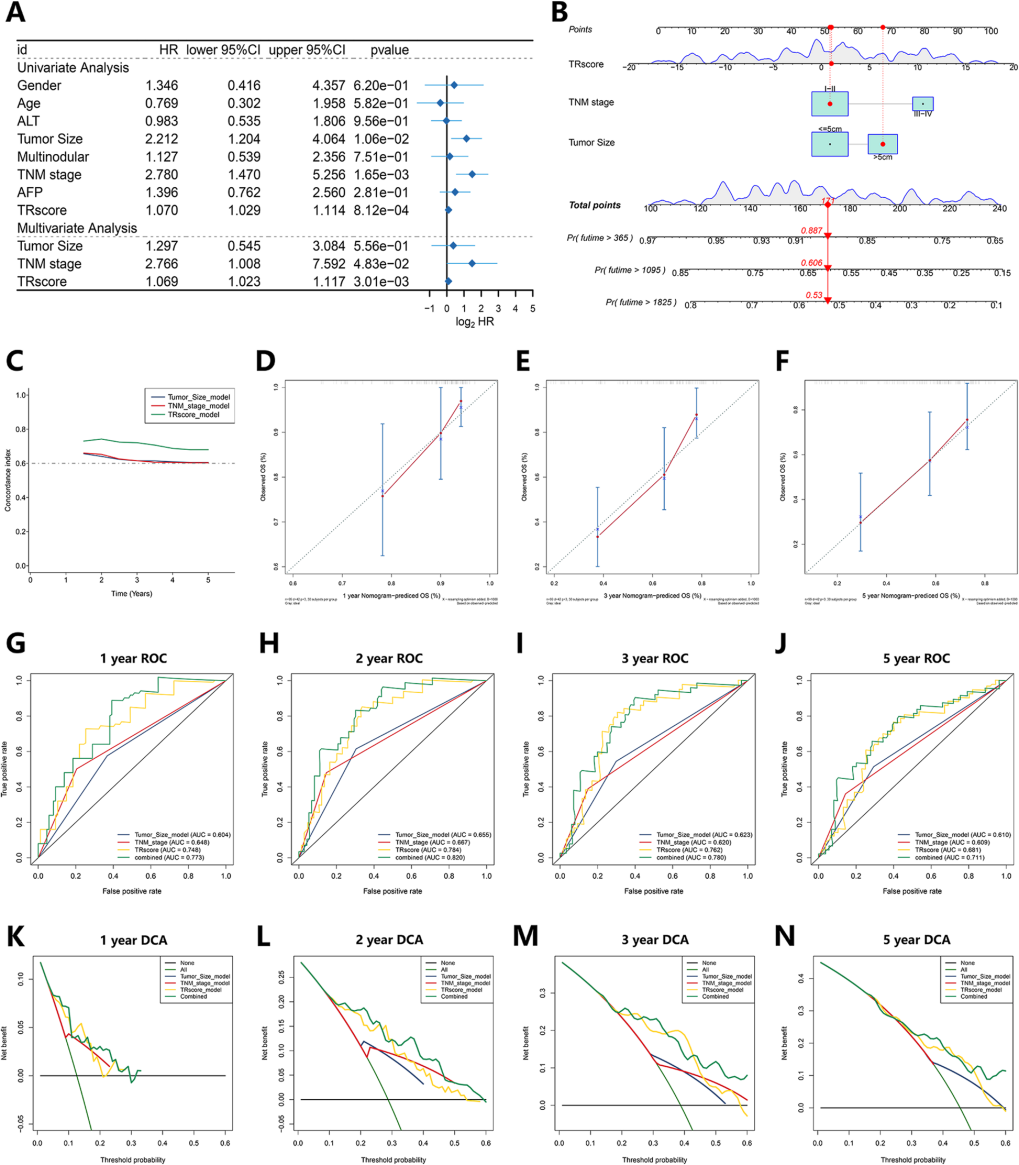
Independent predictive power assessment of the TRscore for the HCC-TACE cohort in the GSE14520 cohort and establishment of the nomogram. **A** Univariate Cox and multivariate Cox regression analyses identify independent predictors of TACE prognosis. **B** Nomogram constructed based on the independent prognostic predictors tumor size, TNM stage and TRscore. **C** The C index shows the agreement between the nomogram-predicted OS and the actual value. **D-F** Calibration curves showing the accuracy of the nomogram in predicting OS at 1 year (**D**), 3 years (**E**), and 5 years (**F**). **G-J** ROC curves showing the predicted reliability of the nomogram at 1 year (**G**), 2 years (**H**), 3 years (**I**) and 5 years (**J**). **K-N** DCA curves showing the clinical benefit of the nomogram in the prediction of OS at 1 year (**K**), 2 years (**L**), 3 years (**M**) and 5 years (**N**)

To assess the ability of the TRscore to independently predict TACE prognosis compared with traditional clinical features, including sex, age, ALT, tumor size, tumor number, TNM stage, and AFP metrics, we used the GSE14520 cohort within the HCC-TACE cohort with univariate and multivariate Cox regression analyses performed on these variables for 99 samples with complete clinical information. The data confirmed that tumor size (HR = 1.297), TNM stage (HR = 2.766) and TR score (HR = 1.069) were independent predictors of prognosis after TACE treatment (Fig. 5A). Based on several independent predictors of tumor size, TNM stage, and TRscore, we constructed a predictive nomogram to quantitatively predict individual TACE prognosis (Fig. 5B). The C-index results showed that among the independent prognostic factors, the TRscore had the best predictive performance (Fig. 5C). Calibration curves for nomograms showed good agreement between predicted 1-, 3-, and 5-year OS and actual observations (Fig. 5D-F). Subsequently, we performed ROC curve analysis to further verify the predictive accuracy of the nomogram. The AUCs of the nomogram for OS at 1, 2, 3, and 5 years were 0.773, 0.820, 0.780, and 0.711, respectively, which were significantly better than the predictive performance of a single independent predictor (Fig. 5G-J). To further assess the guiding value of the nomogram in clinical decision-making, we performed a DCA. We found that nomograms yielded the best net gains at 1, 2, 3, and 5 years compared with a single independent predictor (Fig. 5K-N). The above results suggest that the nomogram has high clinical applicability and excellent predictive ability for predicting the survival probability of HCC patients after TACE.

### Correlation Between Molecular Targeted Drug Sensitivity and TRscore

Molecular targeted drug therapy has also been one of the main treatment options for inoperable HCC patients in recent years. It has been proven to effectively inhibit tumor progression and prolong patient survival. The combination of molecular targeted drugs and TACE has shown good results and development prospects in recent years. Accordingly, we evaluated the sensitivity of the GSE14520 and HCC-TACE cohorts to common molecularly targeted drugs on the Genomics of Cancer Drug Sensitivity (GDSC) website using the half inhibitory concentration (IC50) as a reference standard. We found that the high-TRscore group was more sensitive (*p* < 0.05) to common molecularly targeted chemotherapeutics such as erlotinib, lapatinib, and temsirolimus than the low-TRscore group (Figure S2A-L). The above results suggest that the high-TRscore group is a potentially suitable group for molecular targeted drug therapy, and the combination of molecular targeted drug therapy and TACE in the high TRscore group can achieve more significant therapeutic effects.

### Determination of Key Molecules Related to TACE Response in HCC and Evaluation of Prognostic Predictive Ability

**Fig. 6:**
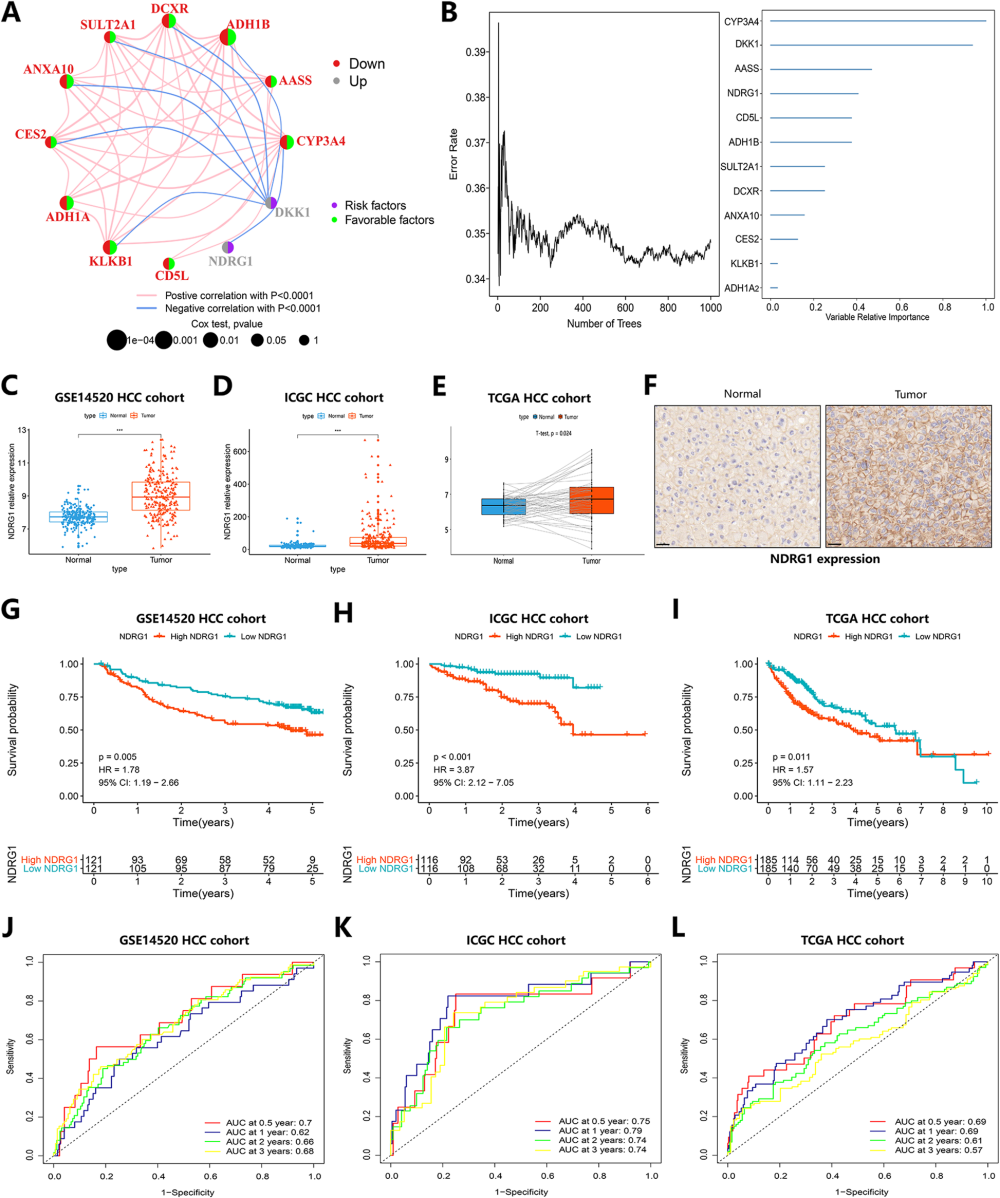
Identification of key molecules associated with TACE response in HCC and their role in prognosis. **A-B** Random forest analysis showing key molecules associated with TACE response in the HCC-TACE cohort of the GSE14520 cohort. **C-E** Boxplots showing the difference in NDRG1 expression between tumors and adjacent normal tissues in the GSE14520 cohort (**C**), ICGC-HCC cohort (**D**), and TCGA-HCC cohort (**E**). **F** Immunohistochemical results showing that the expression of NDRG1 was different in HCC tumor and normal tissues. Ruler: 20 mm. **G-I** Survival curve analysis showing differences in survival prognosis between the high and low NDRG1 groups in the GSE14520 cohort (**G**), ICGC-HCC cohort (**H**) and TCGA-HCC cohort (**I**). **J-L** ROC analysis showing the predictive accuracy of NDRG1 expression in the GSE14520 cohort (**J**), ICGC-HCC cohort (**K**) and TCGA-HCC cohort (**L**)

To further identify the major contributors to the TRscore, the core regulators associated with TACE responsiveness of HCC, we performed random forest analysis on 109 DEGs associated with TACE responsiveness of HCC in the HCC-TACE cohort within the GSE14520 cohort. The results showed that CYP3A4, DKK1, AASS, NDRG1, CD5 L, ADH1B, SULT2A1, DCXR, ANXA10, CES2, KLKB1 and ADH1A were the key influencing factors of TACE response in HCC, of which NDRG1 and DKK1 were risk factors for TACE response (Fig. 6A, B). In previous research reports, DKK1 has been confirmed as a prognostic biomarker for various malignancies [25, 26], and it can well predict the efficacy and prognosis of TACE treatment in HCC patients [27]; however, NDRG1 was not confirmed in previous studies. There is an association between the findings and TACE responsiveness, and its role in the prognosis of HCC patients is not yet clear. Thus, we next focused on exploring the predictive role of NDRG1 in the survival prognosis of HCC.

By analyzing the expression characteristics of NDRG1 in the GSE14520 and HCC cohorts of the ICGC and TCGA databases, we found that NDRG1 exhibited higher expression levels in tumor tissues than in normal tissues (*p* < 0.05) (Fig. 6C-E). Consistently, the results of immunohistochemical analysis also showed that the expression of NDRG1 was significantly increased in tumor tissues compared with adjacent normal tissues (Fig. 6F). The above results suggest that NDRG1 may be a carcinogenic factor in HCC. We then assessed the predictive value of NDRG1 in HCC prognosis. The median NDRG1 expression level was used to divide the high NDRG1 level group and the low NDRG1 level group as the cutoff value. The results of survival prediction analysis showed that the low NDRG1 level group in the GSE14520 dataset showed a more obvious survival advantage than the high NDRG1 level group (*p* = 0.005) (Fig. 6G). The corresponding AUC values reached 0.7, 0.62, 0.66, and 0.68 at 0.5, 1, 2, and 3 years, respectively (Fig. 6J), and the consistent results were also validated in the ICGC-HCC cohort and TCGA-HCC cohort (*p* < 0.05) (Fig. 6H, I), where the AUC values of the ICGC-HCC cohort were 0.75, 0.79, 0.74, and 0.74 at 0.5, 1, 2, and 3 years, respectively (Fig. 6K), and the AUC values of the TCGA-HCC cohort were 0.69, 0.69, 0.61, and 0.57 at 0.5, 1, 2, and 3 years, respectively (Fig. 6L). We also explored the clinical value of NDRG1 in predicting TACE response in HCC patients in GSE104580. The expression level of NDRG1 in TACE no-response tissues was much higher than that in TACE response tissues (Figure S3A). ROC curve, consistency analysis and DCA analysis indicated the superior clinical value of NDRG1 in predicting TACE response in HCC patients (Figure S3B-D). The above results suggest that NDRG1 can effectively predict the survival and prognosis of HCC with excellent predictive ability, as well as the TACE response in HCC patients.

### The Effect of NDRG1 Knockdown on the Proliferation and Migration of HCC Cells

**Fig. 7:**
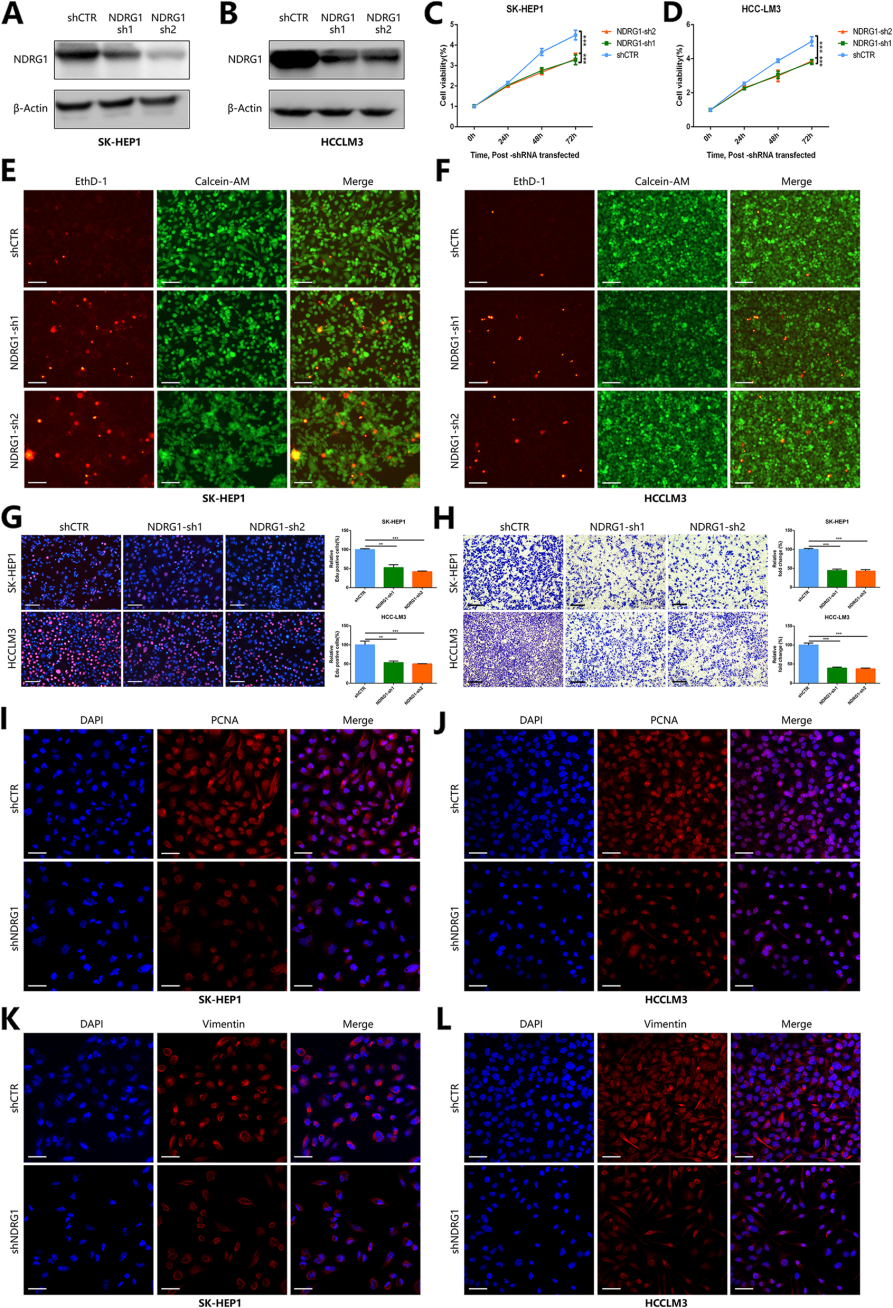
Effects of NDRG1 knockdown on HCC cell proliferation and migration. **A-B** Western blot results showing the knockdown efficiency of NDRG1 shRNA in SK-HEP1 (**A**) and HCCLM3 (**B**) cells. **C-D** CCK8 experiments demonstrate the effect of NDRG1 knockdown on the proliferation of SK-HEP1 (**C**) and HCCLM3 (**D**) cells. **E–F** Live and dead cell staining demonstrated the effect of NDRG1 knockdown on SK-HEP1 (**E**) and HCCLM3 (**F**) cell survival. Ruler: 100 m. **G** EdU experiments showing the effect of NDRG1 knockdown on the proliferation of SK-HEP1 and HCCLM3 cells (**p < 0.01, ***p < 0.001). Ruler: 100 m. **H** Transwell cell migration assay showing the effect of NDRG1 knockdown on the migration of SK-HEP1 and HCCLM3 cells (***p < 0.001). Ruler: 100 m. **I-J** Immunofluorescence experiments showing altered PCNA expression (PCNA red) in SK-HEP1 (**I**) and HCCLM3 cells (**J**) after NDRG1 knockdown. **K-L** Immunofluorescence experiments showing altered expression of Vimentin (Vimentin red) in SK-HEP1 (**K**) and HCCLM3 cells (**L**) after NDRG1 knockdown. Ruler: 200 μm

To further clarify the role of NDRG1 in the oncogenic progression of HCC, we explored the effect of NDRG1 expression on HCC cell proliferation and migration using in vitro cell experiments. First, we constructed NDRG1 knockdown shRNAs with two targets. Western blot results showed that NDRG1 shRNA transfection treatment significantly knocked down NDRG1 expression levels in SK-HEP1 and HCCLM3 cells (Fig. 7A, B). We then examined the effect of NDRG1 knockdown on the proliferation of SK-HEP1 and HCCLM3 cells by the CCK8 assay. The results of CCK8 experiments showed that after NDRG1 knockdown, the proliferation of SK-HEP1 and HCCLM3 cells was significantly inhibited (*p* < 0.001) (Fig. 7C, D). We evaluated the effect of NDRG1 knockdown on HCC cell viability by live/dead cell staining with calcein-AM/EthD-1 double staining. Calcein-AM, which fluoresces green, was used to label live cells, and EthD-1, which fluoresces red, was used to label dead cells. The results showed that the proportion of dead SK-HEP1 and HCCLM3 cells showing red fluorescence significantly increased after NDRG1 knockdown, indicating that NDRG1 knockdown could significantly inhibit the survival of HCC cells (Fig. 7E, F).The EdU experiment results also showed that the EdU-positive rate of SK-HEP1 and HCCLM3 cells in the NDRG1 knockdown group was significantly lower than that in the control group, further confirming the inhibitory effect of NDRG1 knockdown on the proliferation of SK-HEP1 and HCCLM3 cells (*p* < 0.01) (Fig. 7G). The above results suggest that the expression of NDRG1 is closely related to the proliferation of HCC cells and that knockdown of NDRG1 can effectively inhibit the proliferation of HCC cells.

To explore the effect of NDRG1 knockdown on the migration of SK-HEP1 and HCCLM3 cells, we performed transwell cell migration experiments. We found that the migration ability of SK-HEP1 and HCCLM3 cells was significantly inhibited in the NDRG1 knockdown group compared with the control group (*p* < 0.01) (Fig. 7H). To further verify the effect of NDRG1 knockdown on the proliferation and migration of SK-HEP1 and HCCLM3 cells, we also tested the effect of NDRG1 knockdown on the expression of PCNA, a marker protein related to cell proliferation, and Vimentin, a marker protein related to cell migration, by immunofluorescence experiments. The results showed that NDRG1 knockdown effectively suppressed the expression levels of PCNA and Vimentin in SK-HEP1 and HCCLM3 cells (Fig. 7I-L). The above data indicate that NDRG1 expression is closely related to the proliferation, survival and migration of HCC cells and that knockdown of NDRG1 can effectively inhibit the proliferation, survival and migration of HCC cells.

### NDRG1 Inhibition Induced HCC Cells Ferroptosis and Contribute to RLS3-Induced Ferroptosis

**Fig. 8:**
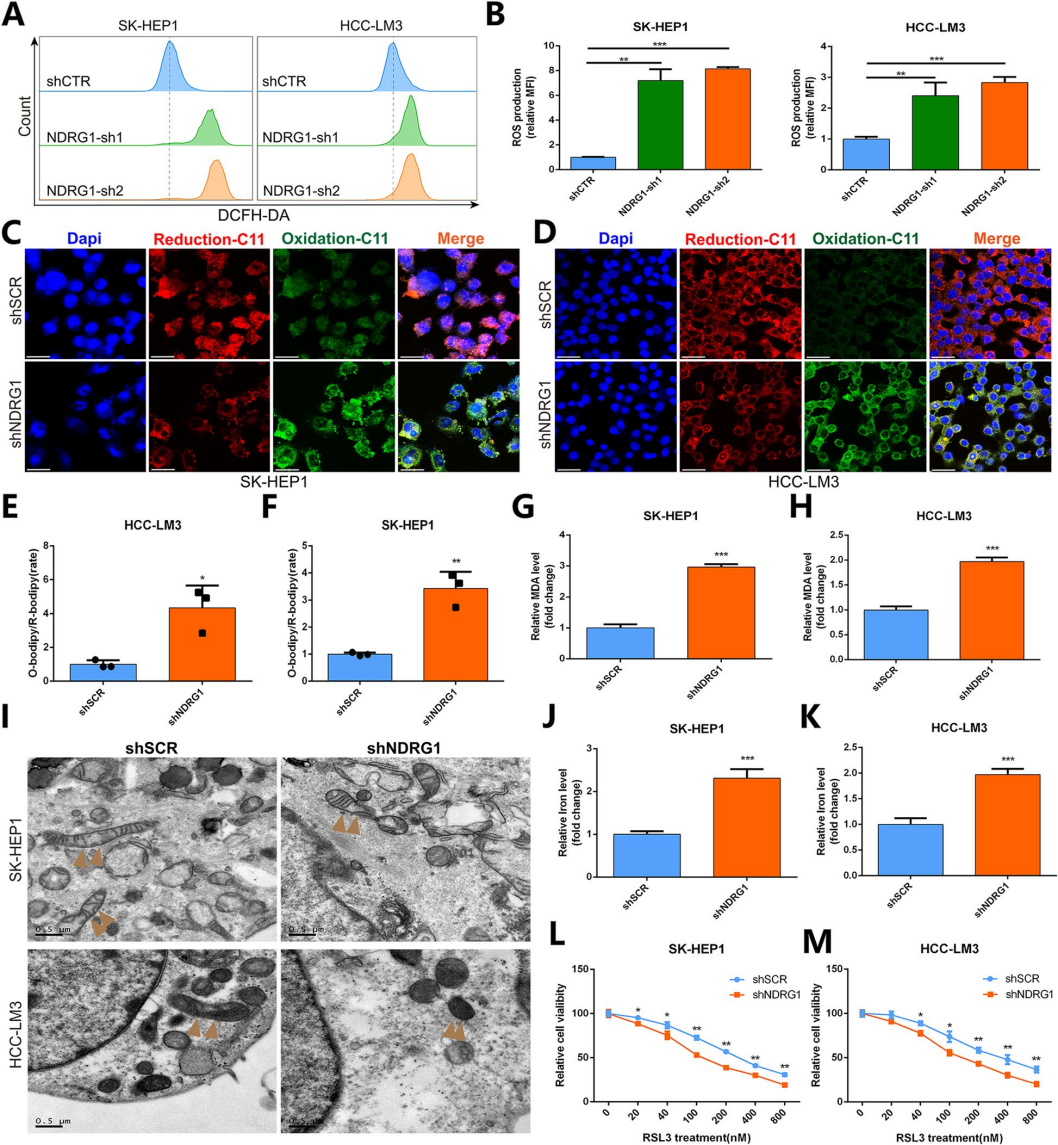
NDRG1 inhibition induced HCC cells ferroptosis and contribute to RLS3-induced ferroptosis. **A-B** FCM and quantification analysis showing increased ROS expression after NDRG1 knockdown in SK-HEP1 and LM3 cells. **C-F** C11-BODIPY (a marker of lipid peroxidation) probe and quantification analysis showing increasing oxidized ROS expression in SK-HEP1 (CE) and LM3 (DF) cells after knockdown of NDRG1. **G-H** Expression levels of MDA after NDRG1 knockdown in SK-HEP1 (**G**) and LM3 (**H**) cells. **I** Representative images of mitochondria within SK-HEP1 and LM3 cells under TEM after NDRG1 knockdown. **J-K** Expression levels of iron ions after knockdown of NDRG1 in SK-HEP1 (**G**) and LM3 (**H**) cells. **L-M** Effect of different concentrations of RSL3 treatment on cell viability of SK-HEP1 and LM3 cells after knockdown of NDRG1

To explore the mechanism by which NDRG1 promotes the proliferation and metastasis of hepatoma cells, we detected ROS expression levels by flow cytometry. The results showed that the ROS expression levels of SK-HEP1 and LM3 cells were significantly up-regulated after knockdown of NDRG1 (Fig. 8A-B). We next detected ferroptosis by determining the amount of lipid peroxides in cellular membranes using BODIPY-C11 probe, and the results showed that the expression level of oxidative C11 was up-regulated after knockdown of NDRG1 (Fig. 8C-F). Therefore, we detected the expression levels of ferroptosis-related indicators (including MDA and iron ions), and the experimental results showed that after knocking down NDRG1, the expression level of MDA (Fig. 8G-H) and iron ions (Fig. 8J-K) in SK-HEP1 and LM3 cells were significantly increased. Previous studies have reported that ferroptosis is primarily characterized by cytological changes, including reduction or disappearance of mitochondrial cristae, rupture of the mitochondrial outer membrane, and mitochondrial membrane condensation. Our experimental results showed under electron microscope that after knockdown of NDRG1, mitochondrial morphology was significantly pyknotic in SK-HEP1 and LM3 cells (Fig. 8I). These above results suggest that NDRG1 expression is closely related to ferroptosis, and NDRG1 knockdown can induce ferroptosis in HCC cells. Ultimately, we treated SK-HEP1 and LM3 cells with different concentrations of RSL3-an inhibitor of glutathione peroxidase 4 (GPX4), and the results showed that knockdown of NDRG1 could induce ferroptosis in the RSL3 pathway (Fig. 8L-M). These data suggest that NDRG1 knockdown can induce ferroptosis in HCC cells and contribute to RLS3-induced ferroptosis.

### The Regulatory Role of NDRG1 in the Growth and Tumor Metastasis of HCC

**Fig. 9:**
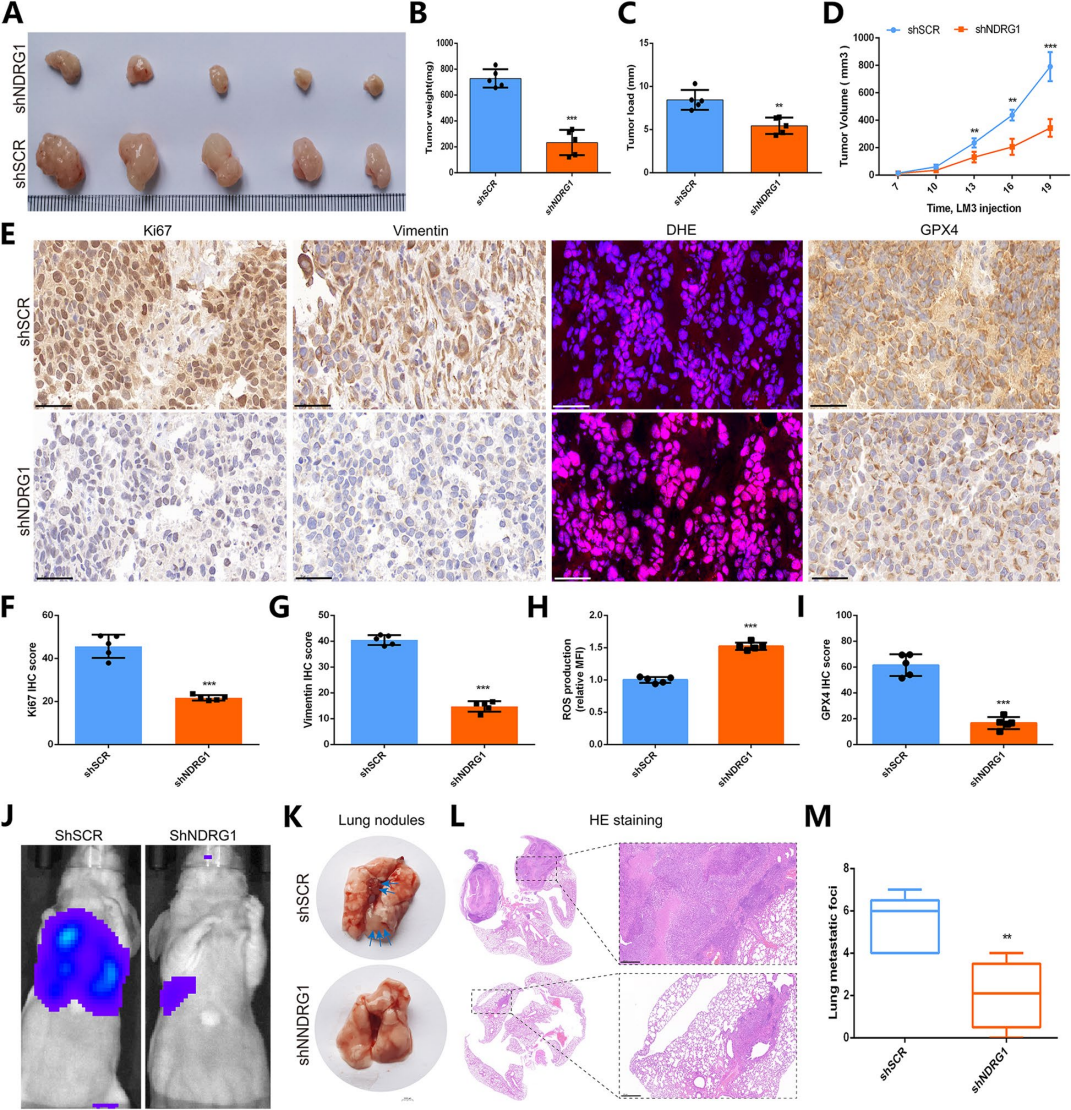
The regulatory role of NDRG1 in the growth and tumor metastasis of HCC.** A-D** A xenograft tumor model of LM3 cells showed that the volume and weight of the tumor were significantly reduced, and the growth rate of the tumor was significantly slowed after NDRG1 knockdown. **E** Immunohistochemical assays revealed that the expression levels of Ki67,Vimentin and GPX4 were inhibited and the expression levels of DHE was upregulated in HCC cells after NDRG1 knockdown. **F-I** The statistical results showed that knockdown of NDRG1 restricted the growth of HCC tumors. **J-M** We constructed a tail vein lung metastasis model, and the results of in vivo imaging (**J**), photography (**K**) and HE staining (**L**) demonstrated that the tumor size and number of HCC lung metastases were markedly decreased after NDRG1 knockdown

To further explore the regulatory role of NDRG1 in the growth and tumor metastasis of HCC, we constructed a xenograft tumor model of LM3 cells and a tail vein lung metastasis model. The results showed that after NDRG1 knockdown, the volume of tumors in LM3 cells was significantly reduced, the weight of the tumors was significantly reduced, and the growth rate of the tumors was significantly slowed (Fig. 9A-D). Immunohistochemical assays showed that knockdown of NDRG1 evidently inhibited the expression levels of Ki67, Vimentin and GPX4, which are proteins related to cell proliferation and migration in HCC tissues. Simultaneously, the expression levels of DHE staining was upregulated in HCC cells, which were closely related to cell ferroptosis (Fig. 9E). The above results showed that knockdown of NDRG1 obviously suppressed the tumorigenic ability of HCC cells in nude mice and induced the ferroptosis of HCC cells. The statistical results also revealed that knockdown of NDRG1 evidently inhibited Ki67, Vimentin and GPX4 expression and increased the level of DHE staining in HCC tissue (Fig. 9F-I). Next, we further explored the effect of NDRG1 expression on the lung metastasis ability of HCC cells through the tail vein lung metastasis model. The results demonstrated that compared with the control group, the tumor size and number of HCC lung metastases were markedly decreased after NDRG1 knockdown (Fig. 9J-M). This result also indicates that the expression of NDRG1 can promote the lung metastasis of HCC cells. These results suggest that NDRG1 functioned as a guardian against ferroptosis to drives tumorgenesis and metastasis in HCC.

## Discussion

As one of the commonly used treatments for HCC, TACE is recommended as the first-choice treatment option for mid-stage BCLC, providing a survival benefit for unresectable HCC [28]. However, due to the heterogeneity of intermediate-stage HCC and the widespread use of TACE beyond the recommendations of the BCLC staging system, patients' tumor response to TACE varies widely, and not all HCC patients benefit from TACE [8]. Therefore, constructing gene signatures and predictive models that can specifically and accurately predict the prognosis of TACE and stratifying patients with the greatest survival benefit after TACE are urgent tasks in the clinical management of HCC. It is also the core strategy of precision oncology.

With the rapid decline in the cost of genome analysis in recent years, the use of accumulated online data to develop new methods to guide disease diagnosis and treatment has become a research hotspot. In this study, we explored the differential gene expression patterns between TACE responders and nonresponders of HCC using the GSE14520 and GSE104580 cohorts and identified 2 TACE-specific associated molecular subtypes of HCC, and the results indicated that Cluster B had worse clinical features of the disease than Cluster A. In contrast, patients in Cluster A showed a better survival advantage. In addition, we developed the TRscore scoring system and successfully validated its specificity and accuracy in predicting prognosis after TACE. The TACE prognostic predictive power of the TRscore was also stable in subgroups stratified by survival status, TNM stage, AFP value, and tumor size, with high TRscore levels mainly expressed in the population who died and those with TNM stage III-IV and AFP > 300 ng/ml. In patients with more severe disease characteristics, such as tumor diameter > 5 cm, the TACE nonresponder group also tended to express higher TRscore levels, indicating that high TRscore levels predict poor prognosis. TRscore is an independent risk factor associated with prognosis after TACE, and to further understand the clinical applicability and utility of TRscore, we constructed a nomogram for predicting 1-, 3-, and 5-year survival probability in HCC patients. The results showed that the nomogram can accurately predict the prognosis of patients with TACE, and it is beneficial to help patients obtain the greatest survival benefit. These findings all highlighted the clinical value of TRscore serving as a reliable prognostic predictor for HCC.

Immunotherapy has played a nonnegligible role in HCC management in recent years. To explore the predictive value of our developed TRscore in response to immunotherapy, we focused on the association between the TRscore and infiltrating immune cells, immune function, and immune checkpoints. Compared with the high TRscore group, the low TRscore group expressed higher levels of immune cell infiltration, such as activated CD8 + T cells, type I CD4 + helper T cells (Th1 cells) and γδ T cells. T cells are key players in antitumor immunity, and activated CD8 + T cells are considered to be the main driver, which can recognize tumor antigens and directly target and kill tumor cells to mediate tumor rejection [29]. Th1 cells are considered to be the most important helper cell type in the anticancer immune response. They can participate in killing tumor cells by secreting cytokines that activate death receptors on the surface of tumor cells and inducing epitope diffusion. They can also activate the cytotoxic function of DCs. Th1 cells eliminate tumor cells in an IFN-γ-dependent manner and provides a source of tumor-associated antigens for killed tumor cells [30]. The protective role of γδT cells during tumor development has been increasingly reported in recent years. γδT cells are key players in cancer immune surveillance, with the ability to sense early changes in the process of tumor cell development [31]. In addition, the low TRscore group showed stronger antigen-presenting cell (APC) costimulation [32], type II IFN (IFNγ) response [33], and immune cytolytic activity (CYT) [34], which are involved in antigen processing and presentation to induce or enhance the immune function of host antitumor immunity. The high-TRscore group expressed higher enrichment levels of metabolic pathways that play a promoting role in tumor development, such as the cell cycle, viral oncogenicity, and epithelial-mesenchymal transition (EMT) [35, 36]. T-cell checkpoint blockade, including anti-PD-L1 and anti-CTLA4, is the preferred strategy for immunotherapy [37]. Our study showed that the low-TRscore group expressed higher levels of immune checkpoints such as PD-L1, PD-L2, CTLA4, IDO1, and GEM than the high-TRscore group, suggesting that when receiving immune checkpoint therapy, the results of the low-TRscore group were more significant. The TRscore can effectively predict immunotherapy response, and patients with low TRscore levels are suitable candidates for immunotherapy. While previous studies revealed that TACE may induce partial immune reconstitution and have a potential synergistic effect with immunotherapy [38], our study also confirmed that patients with low TRscore levels are more likely to respond to TACE. These results suggest that patients with low TRscore levels who receive TACE combined with immunization treatment may achieve better results, which is beneficial to significantly improve the survival and prognosis of patients.

The development of molecularly targeted drugs provides a new therapeutic method for the treatment of HCC, and combination therapy with TACE has also shown good development prospects in recent years, effectively improving the survival of patients [39]. Therefore, we explored the association between the TRscore and molecularly targeted drug therapy through the GDSC database. We found that the sensitivity of multiple molecularly targeted drugs, such as erlotinib, lapatinib, and temsirolimus, was closely related to TRscore, and patients with high TRscores showed higher sensitivity to molecularly targeted drugs. This is an important factor for determining candidates suitable for receiving molecularly targeted therapy, opening up new prospects for optimizing individualized treatment of patients.

Ferroptosis is a newly discovered mode of programmed cell death characterized by iron-dependent lipid peroxidation and accumulation of reactive oxygen species (ROS) [40–43]. Previous studies have found that ferroptosis plays an important role in the progression and treatment of HCC, and further excavation of the mechanism will help to develop new therapeutic strategies, however, the specific mechanism is still unclear. Our study discovered that knockdown of NDRG1 can increase ROS and iron accumulation, therefore, we believe that it can act as a suppressor of ferroptosis to promote the occurrence and metastasis of HCC. More interestingly, we found that knockdown of NDRG1 increased RSL3-induced ferroptosis. Previous studies reported that RSL3 was an inhibitor of glutathione peroxidase 4 (GPX4), so we speculated that NDRG1 might act by affecting the expression of GPX4. Consequently, we believe that NDRG1 may act as a guardian against ferroptosis to drive tumorigenesis and metastasis in HCC.

This study also identified 12 central characteristic genes related to TACE response in HCC and revealed that DKK1 and NDRG1 were risk factors in TACE response. Although DKK1 has been proven to be a good predictor of TACE treatment efficacy and prognosis in HCC patients in previous studies [27], NDRG1 has not been found to be associated with TACE responsiveness in previous studies, and it has a role in the prognosis of HCC patients. The effects of DKK1 are unclear, so we next focused on the role of NDRG1 as a TACE response-related gene in HCC progression and prognosis. NDRG1, a member of the NDRG protein family, has been shown in previous studies to have anticarcinogenic and antimetastatic effects in cancers, including breast, prostate, and colorectal cancer [44]. It promotes tumorigenesis of bladder cancer, HCC and esophageal cancer [45–47]. In our study, through the online data analysis of the GSE14520, ICGC-HCC and TCGA-HCC cohorts, as well as the analysis of collected HCC case data, the results showed that the expression level of NDRG1 in tumor tissues was higher than that in normal tissues. Compared with patients with low expression of NDRG1, patients with high expression of NDRG1 have a worse survival prognosis, suggesting that NDRG1 may be a carcinogenesis-related factor of HCC, and the expression level is closely related to the poor prognosis of patients. Subsequent cell experiments further revealed that knockdown of NDRG1 can effectively inhibit the cloning, proliferation and migration of HCC cells, confirming that NDRG1 is an oncogenic factor of HCC and plays a promoting role in the occurrence and development of tumors. Next, we examined the effect of knocking down NDRG1 on ferroptosis in HCC cells and certificated that NDRG1 knockdown may induce ferroptosis and contribute to RLS3-induced ferroptosis in HCC cells. Then, we constructed a xenograft tumor model of LM3 HCC cells, and the results showed that knockdown of NDRG1 significantly inhibited the tumorigenic ability of HCC cells in nude mice and induced the ferroptosis of HCC cells. The results of the tail vein lung metastasis model further revealed that the tumor size and tumor number of HCC lung metastasis were obviously decreased after NDRG1 knockdown, which also signified that the expression of NDRG1 could promote the lung metastasis of HCC cells. The above results confirm that NDRG1 may be a key factor in the regulation of HCC tumor progression and may act as a guardian against ferroptosis to drive tumorgenesis and metastasis in HCC.

Our study combines the bioinformatics analysis of the online database with experiments both in vivo and in vitro, which provides a reliable way for the screening of potential novel carcinogenic markers for HCC. However, due to the large amount of bioinformatics analysis carried out in this project, there are inevitably some limitations related to bioinformatics analysis [48–50]. First of all, our study was based primarily on bioinformatics analysis, and the results obtained from online database required a large clinical cohort for further validation with solid clinical specimens, such as collecting more clinical cohorts containing information of TACE response and non-response to improve the stability of the analysis results, and the clinical feasibility of the TRscore in predicting the response of chemotherapy immunotherapy required further test in subsequent clinical trials. In addition, the study was conducted using a retrospective design rather than a prospective design, and the obtained data may be confounded by some factors; thus, it is necessary to carry out prospective clinical trials and mechanism exploration studies in the future to further validate the current results. Moreover, the oncogenic role of NDRG1 in HCC requires more experiments to validate the results, including a conditional knockout mouse model, and the mechanism of NDRG1 in HCC also needs to be further explored.

## Conclusion

TACE response-related molecular subtypes of HCC can effectively predict TACE prognosis with stable prediction performance. The TRscore can specifically and accurately predict the prognosis of TACE and is closely related to the clinical characteristics of the disease. High TRscore levels predict poor prognosis. TRscores help to identify candidates suitable for immunotherapy and molecularly targeted drug therapy. Individuals with low TRscores have a stronger immune response ability and are more likely to benefit from immunotherapy, while individuals with high TRscores are more likely to benefit from molecularly targeted drugs and are more sensitive to medications. The nomogram constructed based on the TRscore can effectively predict the prognosis of TACE in HCC patients with good predictive accuracy. The TACE response-related core molecule NDRG1 can accurately and effectively predict the survival and prognosis of HCC, and high NDRG1 expression indicates poor prognosis. Knockdown of NDRG1 inhibits the survival, proliferation and migration of HCC cells, and NDRG1 plays a promoting role in tumor progression. Through the integration and comprehensive analysis of the genomic data of HCC patients, our study shows that the TACE response-related molecular subtypes and TRscore of HCC can be used as effective predictive tools for the response and prognosis of HCC patients treated with TACE. The findings of this study provide the possibility to identify patients with the greatest survival benefit after TACE, improve patient prognosis, and provide new ideas for the development of individualized treatment strategies.

## Supplementary Information

**Additional file 1: Figure S1.** A flowchart depicting the research process.

**Additional file 2: Figure S2.** A-L Correlation of the TRscore of the HCC-TACE cohort in the GSE14520 cohort with the sensitivity to common molecularly targeted drugs.

**Additional file 3: Figure S3.** The predictive performance of NDRG1 in predicting the TACE response of HCC patients. A Expression characteristics of NDRG1 in TACE responders and nonresponders. B The ROC curve showing the predictive reliability of NDRG1 in predicting the TACE response of HCC patients. C consistency analysis of NDRG1 expression in predicting the TACE response of HCC patients. D DCA of NDRG1 in predicting the TACE response of HCC patients.

## Data Availability

The data and materials used to support the findings of this study are available from the corresponding author upon request.

## Abbreviations

PLC: Primary liver cancer
HCC: Hepatocellular carcinoma
TACE: Transcatheter arterial chemoembolization
TME: Tumor microenvironment
ICI: Immune checkpoint inhibitor
ROC: Receiver operating characteristic curve
DCA: Decision curve analysis
PCA: Principal component analysis
TRscore: TACE response score
DEGs: Differentially expressed genes
AFP: Alpha fetoprotein
ALT: Alanine aminotransferase
PD-1: Programmed cell death protein 1
PD-L1: Programmed cell death 1 ligand 1
KEGG: Kyoto Encyclopedia of Genes and Genomes
GO: Gene ontology
AUC: Area under the curve
GSVA: Gene set variation analysis
GSEA: Gene set enrichment analysis
OS: Overall survival
PVDF: Polyvinylidene fluoride
PBS: Phosphate-buffered saline
IF: Immunofluorescence
DAPI: 4',6-Diamidino-2-phenylindole
