# Supplementary figures and images for: TACE responser NDRG1 acts as a guardian against ferroptosis to drive tumorgenesis and metastasis in HCC
Source: Biol Proced Online. 2023 May 19;25:13. doi: 10.1186/s12575-023-00199-x (PMC10197860; doi:10.1186/s12575-023-00199-x)

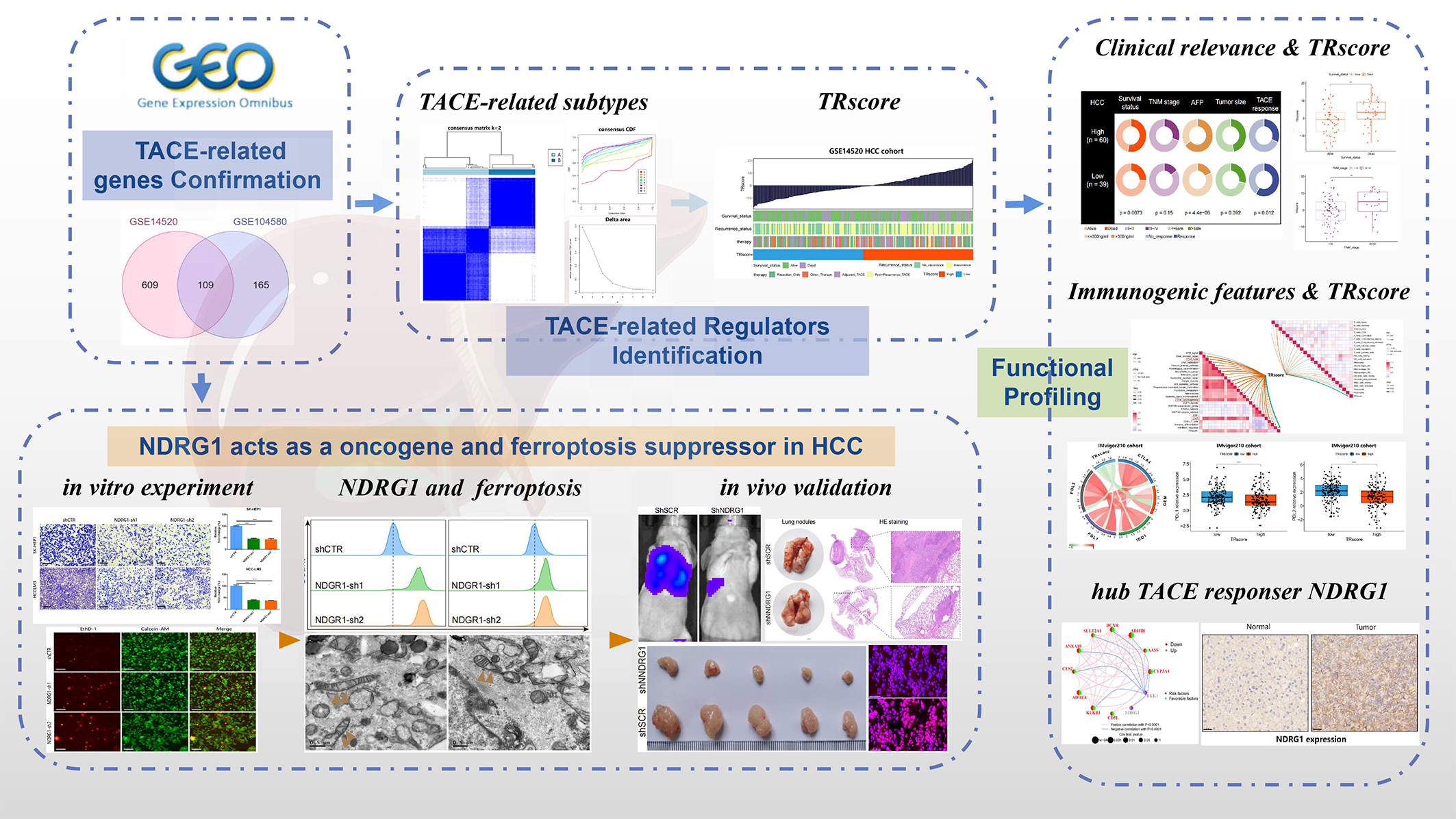

Supplement: Supplementary file 1 — Additional file 1: Figure S1. A flowchart depicting the research process. [file 12575_2023_199_MOESM1_ESM.tif]

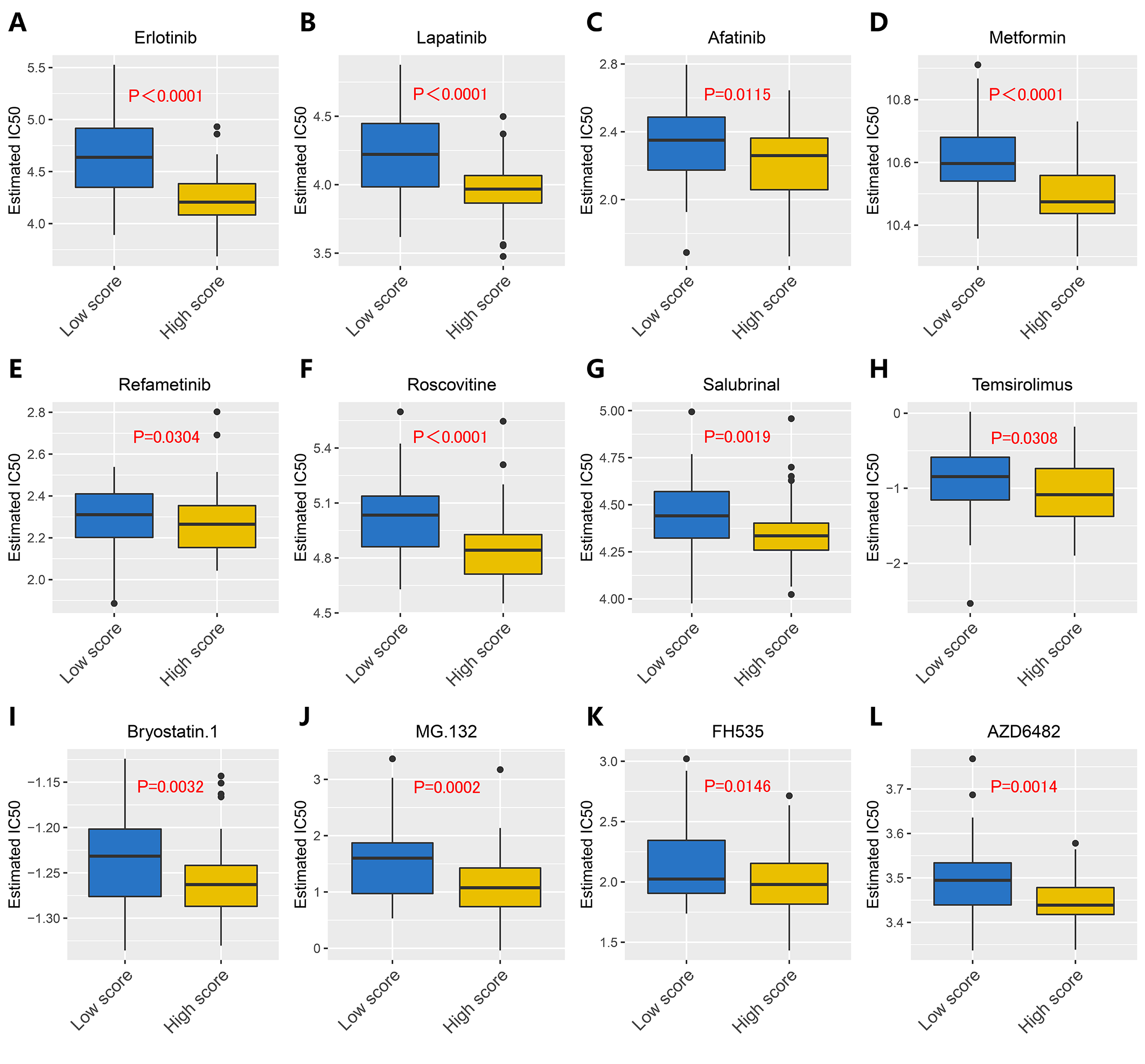

Supplement: Supplementary file 2 — Additional file 2: Figure S2. A-L Correlation of the TRscore of the HCC-TACE cohort in the GSE14520 cohort with the sensitivity to common molecularly targeted drugs. [file 12575_2023_199_MOESM2_ESM.tif]

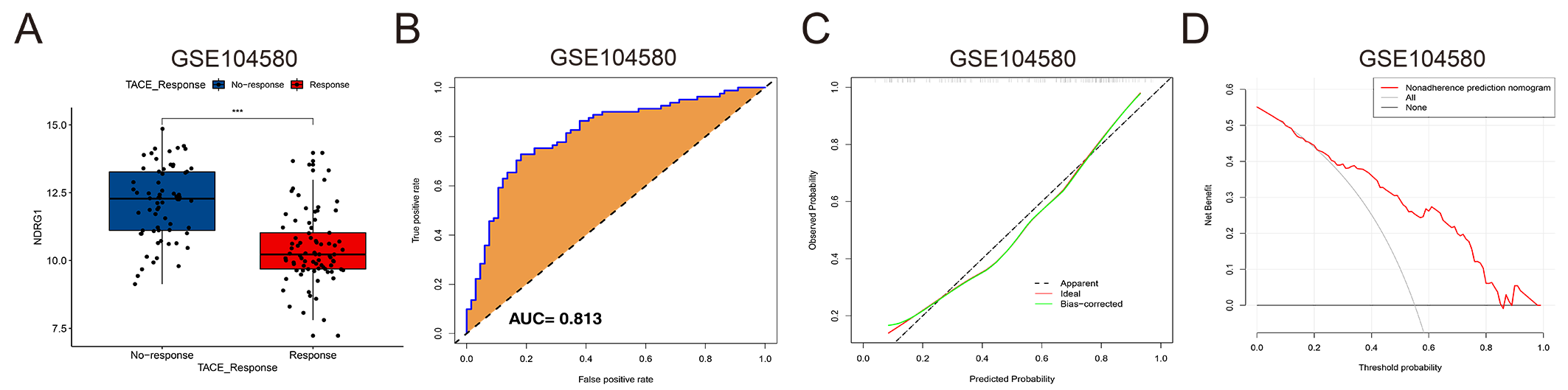

Supplement: Supplementary file 3 — Additional file 3: Figure S3. The predictive performance of NDRG1 in predicting the TACE response of HCC patients. A Expression characteristics of NDRG1 in TACE responders and nonresponders. B The ROC curve showing the predictive reliability of NDRG1 in predicting the TACE response of HCC patients. C consistency analysis of NDRG1 expression in predicting the TACE response of HCC patients. D DCA of NDRG1 in predicting the TACE response of HCC patients. [file 12575_2023_199_MOESM3_ESM.tif]
